# Dipeptidyl peptidase IV, aminopeptidase N and DPIV/APN-like proteases in cerebral ischemia

**DOI:** 10.1186/1742-2094-9-44

**Published:** 2012-02-28

**Authors:** Peter Röhnert, Werner Schmidt, Patrick Emmerlich, Alexander Goihl, Sabine Wrenger, Ute Bank, Karsten Nordhoff, Michael Täger, Siegfried Ansorge, Dirk Reinhold, Frank Striggow

**Affiliations:** 1 KeyNeurotek Pharmaceuticals AG, Leipziger Str. 44, D-39120 Magdeburg, Germany; 2 European Screeningport GmbH, Schnackenburgallee 114, D-22525 Hamburg, Germany; 3 Department of Neurodegeneration and Intervention Strategies, German Center for Neurodegenerative Diseases (DZNE), Leipziger Str. 44, D-39120 Magdeburg, Germany; 4 Institute of Molecular and Clinical Immunology, Otto-von-Guericke-University, Medical Faculty, Leipziger Str. 44, D-39120 Magdeburg, Germany; 5 IMTM GmbH, Leipziger Str. 44, D-39120 Magdeburg, Germany

**Keywords:** Cerebral schemia, Stroke, Middle cerebral artery occlusion, DPIV, Aminopeptidase N

## Abstract

**Background:**

Cerebral inflammation is a hallmark of neuronal degeneration. Dipeptidyl peptidase IV, aminopeptidase N as well as the dipeptidyl peptidases II, 8 and 9 and cytosolic alanyl-aminopeptidase are involved in the regulation of autoimmunity and inflammation. We studied the expression, localisation and activity patterns of these proteases after endothelin-induced occlusion of the middle cerebral artery in rats, a model of transient and unilateral cerebral ischemia.

**Methods:**

Male Sprague-Dawley rats were used. RT-PCR, immunohistochemistry and protease activity assays were performed at different time points, lasting from 2 h to 7 days after cerebral ischemia. The effect of protease inhibitors on ischemia-dependent infarct volumes was quantified 7 days post middle cerebral artery occlusion. Statistical analysis was conducted using the *t*-test.

**Results:**

Qualitative RT-PCR revealed these proteases in ipsilateral and contralateral cortices. Dipeptidyl peptidase II and aminopeptidase N were up-regulated ipsilaterally from 6 h to 7 days post ischemia, whereas dipeptidyl peptidase 9 and cytosolic alanyl-aminopeptidase were transiently down-regulated at day 3. Dipeptidyl peptidase 8 and aminopeptidase N immunoreactivities were detected in cortical neurons of the contralateral hemisphere. At the same time point, dipeptidyl peptidase IV, 8 and aminopeptidase N were identified in activated microglia and macrophages in the ipsilateral cortex. Seven days post artery occlusion, dipeptidyl peptidase IV immunoreactivity was found in the perikarya of surviving cortical neurons of the ipsilateral hemisphere, whereas their nuclei were dipeptidyl peptidase 8- and amino peptidase N-positive. At the same time point, dipeptidyl peptidase IV, 8 and aminopeptidase N were targeted in astroglial cells. Total dipeptidyl peptidase IV, 8 and 9 activities remained constant in both hemispheres until day 3 post experimental ischemia, but were increased (+165%) in the ipsilateral cortex at day 7. In parallel, aminopeptidase N and cytosolic alanyl-aminopeptidase activities remained unchanged.

**Conclusions:**

Distinct expression, localization and activity patterns of proline- and alanine-specific proteases indicate their involvement in ischemia-triggered inflammation and neurodegeneration. Consistently, IPC1755, a non-selective protease inhibitor, revealed a significant reduction of cortical lesions after transient cerebral ischemia and may suggest dipeptidyl peptidase IV, aminopeptidase N and proteases with similar substrate specificity as potentially therapy-relevant targets.

## Background

Focal cerebral ischemia is accompanied by marked inflammatory reactions in the affected brain regions, initiated by microglia and astrocytes activation and the generation of inflammatory mediators such as pro-inflammatory cytokines and free oxygen radicals [1,2]. Moreover, during focal cerebral ischemia, the local disruption of the blood brain barrier leads to an invasion of reactive polymorphonuclear neutrophils from the periphery into the brain [3-5]. In addition to the breakdown of oxygen und substrate supply due to vessel occlusion, invading immune competent cells and the release of neurotoxic mediators appear to be involved in the acceleration of neuronal cell damage [6]. The activation of microglia/macrophages and astrocytes as well as the infiltration of leukocytes into the ischemic area is reported to maintain for hours and days after the initial ischemic insult [7]. Therefore, anti-inflammatory treatments of the ischemic brain may constitute a therapeutic option to target delayed pathophysiological processes and to manage neuronal degeneration and cerebral damage.

Peptidases like dipeptidyl peptidase IV (DPIV, CD26, E.C. 3.4.14.5) and aminopeptidase N (APN, CD13, E.C. 3.4.11.2) are known to regulate a variety of biological processes related to inflammation such as T cell activation, immune responses and inflammation-related diseases [8-11]. DPIV, a 110 kD type II transmembrane glycoprotein, identical with the T cell antigen CD26, belongs to the group of post-proline dipeptidyl aminopeptidases, consisting of five DPIV gene family proteases, i.e. DPIV, fibroblast activation protein (FAP), DP8, DP9, and DPII (E.C.3.4.14.2) [10-13].

DPIV catalyzes the release of N-terminal dipeptides from oligo- and polypeptides, preferentially with a proline, hydroxyproline or, although with lower efficiency, alanine in the penultimate position [8-11]. The unique substrate specificity of DPIV and DPIV-like enzymes underlies their key role in the catabolism of a number of chemo- and cytokines, neuropeptides, immunopeptides and peptide hormones containing a X-Pro or X-Ala amino terminal sequence, e.g. CXCL12, substance P, neuropeptide Y, peptide YY, enterostatine, glucose-dependent insulinotropic polypeptide (GIP), and glucagon-like peptide-1 (GLP-1) [14-16]. Recently, it has been shown that an additional binding site in the central pore of DPIV is responsible for the cellular effects of ligands of this enzyme with respect to growth regulation and cytokine production [17].

APN, identical with the myeloid linage antigen CD13, is a 150 kD type II transmembrane metalloprotease. It belongs to the family of zinc-dependent aminopeptidases, found in different subcellular organelles, in the cytoplasm and as integral membrane proteins. APN is responsible for the hydrolysis of neutral amino acids from the N-terminus of oligopeptides. The peptidase stops peptide hydrolysis, if a proline appears in the second position of the N-terminal sequence, thereby generating potentially DPIV-susceptible substrates. This mechanism may underlie a co-operative mode of action between APN and DPIV [18]. APN has been shown to be involved in the degradation of several neuropeptides, angiotensins, cytokines and immunomodulatory peptides. APN also contributes to extracellular matrix degradation and antigen processing [18,19].

Recent evidence points also towards a role of the APN-like peptidase cytosolic alanyl-aminopeptidase (cAAP) in the immune response. Expression of cAAP mRNA was identified in CD4+, CD8+, Th1, Th2 and Treg (CD4+ CD25+) T cell subpopulations [20].

DPIV, APN and the DPIV- and APN-like peptidases are constitutively expressed in different brain regions [21-25], indicating an important role in brain physiology. Furthermore, it has been postulated that this group of peptidases might be involved in neurodegenerative processes due to different acute CNS insults such as a stroke or traumatic brain injury [26,27].

To further examine the function of DPIV, APN and/or DPIV-/APN-like peptidases in ischemia-dependent neurodegeneration, we have studied these target peptidases in an *in vivo *model of transient, unilateral cerebral ischemia due to endothelin-induced occlusion of the middle cerebral artery (eMCAO). Using RT-PCR and enzymatic assays, we analyzed the temporal pattern of expression and peptidase activity of dipeptidyl peptidase II (DPII), DPIV, dipeptidyl peptidase 8 (DP8), dipeptidyl peptidase 9 (DP9), APN and cAAP in ipsilateral (infarct) and contralateral (control) cortices after eMCAO and transient cerebral ischemia. In addition, the cell-type-specific localization patterns of DPIV, DP8 and APN in neurons, astroglia, immune-reactive microglia cells and activated macrophages were characterized by multi-labeling immunohistochemistry at defined time points post eMCAO.

IPC1755, a non-selective inhibitor of DPIV/DPIV-like and APN/cAAP protease activities, was able to decrease cortical, eMCAO-induced lesion sizes. This effect was also observed, if the compound was applied exclusively post insult. In addition, distinct expression, localization and activity patterns of DPIV, APN and DPIV-/APN-like proteases indicate a crucial role of these targets in ischemia-induced inflammation and neurodegeneration. Hence, DPIV and/or APN inhibitors may provide a basis for the design of new and more efficient therapeutic strategies against the deleterious consequences of cerebral ischemia.

## Results

### mRNA expression of DPII, DPIV, DP8, DP9, APN and cAAP at different time points after eMCAO

**Figure 1:**
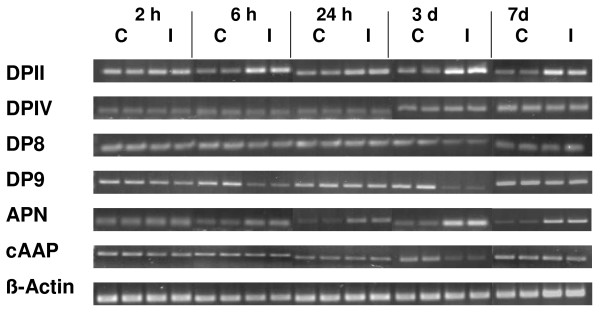
**Rat brain mRNA expression of DPII, DPIV, DP8, DP9, APN and cAAP after focal cerebral ischemia due to eMCAO**. Protease and β-actin mRNAs of ipsilateral (infarct, I) and corresponding contralateral (control, C) hemispheres are represented. Time points post eMCAO as indicated. One representative of four independent experiments is shown.

Sprague-Dawley rats were exposed to a transient focal cerebral ischemia due to eMCAO. 2 h, 6 h, 24 h, 3 d, and 7 d post eMCAO, cortices of the affected ipsilateral hemisphere and the non-ischemic contralateral side were separately collected and analyzed by qualitative RT-PCR. Contralateral cortical tissue probes were used as internal controls. Data obtained from contralateral, non-ischemic cortices were similar to those using the corresponding brain area of untreated or sham-operated adult rat brains, not exposed to eMCAO (not shown). As shown in Figure 1, all peptidases considered in here were expressed in both hemispheres. Nevertheless, we found increased levels of DPII and APN mRNA expression in the ipsilateral (ischemic) hemisphere, lasting from 6 hours to day 7 post eMCAO. In contrast, mRNA expression of DP9 was diminished in the same region at 6 hours and day 3. A reduced mRNA content was also observed for cAAP at day 3 after eMCAO. DPIV and DP8 mRNA levels remained constant in both hemispheres at all time points analyzed (Figure 1).

### Immunohistochemical detection of DPIV, DP8 and APN after eMCAO

In order to characterize the cellular localization of DPIV, DP8 and APN after eMCAO, we performed a multi-labeling immunohistochemistry approach using protease-specific antibodies and cell-specific markers for neurons, astroglia, immune-reactive microglia cells or activated macrophages at defined time points post eMCAO.

Unilateral ischemic brain damage induced by eMCAO caused an infarction in the lateral parts of the frontal cortex extending through the parietal and insular cortex rostrally and through temporal and occipital cortex caudally. In addition, there was evidence of infarction within dorsolateral portions of the caudate nucleus. To analyze the localisation of DPIV, DP8 and APN, the lateral part of the frontal cortex ipsilateral to the infarct area including the penumbra was selected. Corresponding cortical areas of the contralateral hemisphere were chosen as internal control.

**Figure 2:**
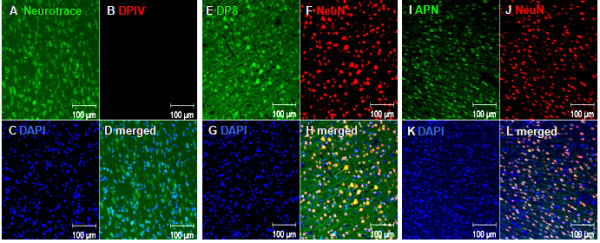
**Immunohistochemical localization of DPIV, DP8 and APN in contralateral cortices 7 days after eMCAO**. Co-stainings were performed using the neuronal markers Neurotrace (A) or NeuN (F, J). DPIV (B), DP8 (E) and APN (I) -linked fluorescences were visualized by confocal laser scan microscopy. DAPI was used to stain cell nuclei (C, G, K, blue fluorescence). Furthermore, merged fluorescence images are shown (D, H, L). Contralateral hemispheres were not exposed to eMCAO. Note, DPIV immunostaining was not found under these conditions (see B). Similar results were obtained in untreated or sham-operated animals as well as 3 days after eMCAO in the contralateral cortex (not shown). Scale bars = 100 μm.

**Figure 3:**
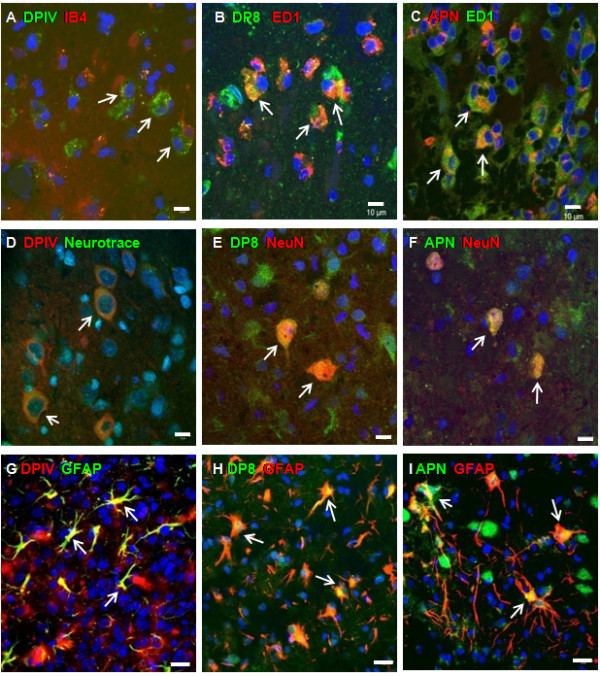
**Immunohistochemical staining of DPIV, DP8 and APN in ipsilateral cortices at different time points after eMCAO**. Confocal fluorescence images were captured either 3 (A-C) or 7 days (D-I) post eMCAO. DAPI was used to stain cell nuclei (A-I, blue fluorescence). At day 3, the time point when microglia activation peaked, DPIV (A, green florescence) was co-stained with IB4 (red) to indentify immunoreactive microglia, whereas DP8 (B, green) and APN (C, red) were co-labeled with ED1 (B, red; C, green), a marker of reactive macrophages. Co-localization of DPIV, DP8 or APN labeling with IB4 (activated microglia) and ED1 (active microglia/macrophages), respectively, is visualized by merged fluorescence signals (A-C, yellow, arrows). At day 7, DPIV (D, G, red), DP8 (E, H, green) and APN (F, I, green) were co-stained with Neurotrace targeting neuronal prerikarya (D, green) or NeuN, labeling neuronal nuclei (E, F, red). Alternatively, DPIV, DP8 and APN were co-labeled with GFAP, expressed in astroglial cells (G, green; H and I, red). Co-localization of each protease with the respective cell markers is seen (D-I, yellow, arrows). Scale bars = 10 μm (A-F) and 20 μm (G-I).

In the non-ischemic, contralateral cortex as well as in other contralateral brain areas, DPIV immunoreactivity was generally not observed (Figure 2A-D). DP8 (Figure 2E-H) and APN (Figure 2I-L) were co-localized with NeuN immunoreactivity, demonstrating their constitutive localisation in neurons of the contralateral cortex. In the same brain area, DP8 and APN were not co-labeled with GFAP immunoreactivity, a marker of astroglial cells, or IB4, specific for immunoreactive microglia (not shown). Three days after eMCAO, DPIV was found to be co-localized with IB4-positive microglia in the infarct area of the ipsilateral cortex (Figure 3A). In parallel, DP8 and APN matched ED1-linked fluorescence of reactive microglia/macrophages (Figure 3B, C). In addition, a small number of surviving, NeuN-positive neurons in the infarct core of the ipsilateral cortex were co-stained with DP8 and APN immunoreactivity, whereas DPIV-positive neurons were not identified at day 3 (not shown). Thus, DP8 and APN localization can be addressed to the perikarya of surviving neurons in the ipsilateral cortex.

Seven days after the induction of eMCAO, surviving, NeuN-positive neurons in the ischemic cortical penumbra remained DP8- and APN-positive (Figure 3E,F). Furthermore, we were able to identify neuron-specific DPIV immunoreactivity, tagged with Neurotrace, a marker of neuronal perikarya (Figure 3D). In parallel, DPIV, DP8 and APN were also found to be co-stained with GFAP immunoreactivity (Figure 3G-I), demonstrating the presence of these proteases in astroglial cells. At day 7 post eMCAO, DPIV, DP8 and APN immunofluorescence was not observed in microglia and macrophages anymore (not shown).

### DPIV/DPIV-like and APN/APN-like protease activities in the brain

To determine the distribution of the distinct proteases of interest in cerebral homogenates, we established an enzyme activity competition assay utilizing selective DPII, DPIV, DP8/9 or cAAP inhibitors. Based on this, specific protease activities were calculated by a non-linear regression analysis (see Methods for details).

**Figure 4:**
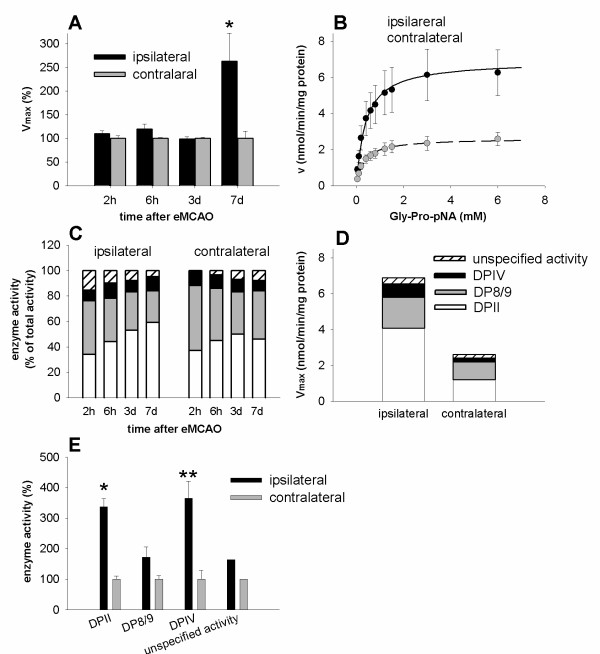
**DPIV and DPIV-like protease activities after transient cerebral ischemia**. (A) Relative V_max _of DPIV/DPIV-like activity in adult rat cortices of ipsilateral (infarct, black bars) or contralateral (control, gray bars) hemispheres at different time points after eMCAO. A pronounced V_max _increase in the ipsilateral cortex compared to the contralateral cortex was detectable 7 day post insult (+165%, for details, see text). At earlier time points, differences between both hemispheres were not found (n = 5). (B) Michaelis-Menten kinetics of total DPIV/DPIV-like activity in infarct (solid line) and control cortices (dashed line) 7 days post eMCAO. Ipsilateral and contralateral cortices revealed similar K_m _values, i.e. ipsilateral: K_m _= 380 ± 40*μ*M; n = 5; contralateral: K_m _= 330 ± 20*μ*M; n = 5, whereas V_max _differed significantly (*p *= 0.025, *t*-test), i.e. ipsilateral: 6.9 ± 1.5 nmol/min/mg protein, n = 5; contralateral: 2.6 ± 0.4 nmol/min/mg protein, n = 5. (C) Relative fractional contribution of DPII, DP8/9 and DPIV activity to total DPIV-like protease activity (Gly-Pro-pNA cleavage) in the ipsilateral (infarct, left bar group) and contralateral (control, right bar group) cortex at different time points after eMCAO (pH **7**.5). (D) V_max _of DPII, DPIV and DP8/9 in the ipsilateral and contralateral cortex. (E) Relative activities of DPII, DPIV or DP8/9 in ipsilateral (black bars) or contralateral control (gray bars) cortices 7 days post eMCAO. Data are means ± SD from 5 independent experiments. *p = 0.0001; **p = 0.0004 (*t*-test).

Kinetic studies of DPIV and DPIV-like proteases according to Michaelis-Menten were performed using homogenates of contralateral (control) or ipsilateral (infarct) cortices prepared from adult rats at different time points after eMCAO. As shown in Figure 4A, DPIV/DPIV-like activity remained unaffected and almost equally distributed in both hemispheres until day 3 post insult. However, maximum total DPIV/DPIV-like activity (V_max_) was significantly increased (+165%) in the ipsilateral cortex compared to the corresponding contralateral cortex 7 days post eMCAO (*p *= 0.025, Figure 4A, B). V_max _of DPIV/DPIV-like activity was 6.9 ± 1.5 nmol/min/mg protein (n ± 5) and 2.6 ± 0.4 nmol/min/mg protein (n = 5) in the ipsilateral and contralateral cortex, respectively. In contrast to V_max_, Michaelis-Menten constants (K_m_) of protease-catalyzed substrate hydrolysis remained unchanged in both hemispheres (ipsilateral: K_m _= 380 ± 40*μ*M, n = 5; contralateral: K_m _= 330 ± 20*μ*M, n = 5, Figure 4B).

Subsequently, we studied the fractional distribution of DPII, DPIV and DP8/9 with respect to the total DPIV/DPIV-like activity in contra- and ipsilateral cortices from 2 h until 7 days post eMCAO. A small fraction of DPIV/DPIV-like activity was continuously detectable in adult cortical homogenates, including the contralateral hemisphere, at all time points post eMCAO considered in here. 2 h post eMCAO, DPII-specific activity was 34 ± 4.3% of total DPIV/DPIV-like activity in the ipsilateral (infarct) cortex versus 39 ± 3.3% contralateral (Figure 4C). In parallel, DP8/9 activity was 42 ± 5.6% ipsilateral versus 49 ± 3.7% contralateral. At the same time point, a small, but similar degree of DPIV activity was detectable ipsilateral (8.5 ± 5.3%) and contralateral (11 ± 5.8%, Figure 4C).

At day 7 post eMCAO, the fractional distribution of different DPIV/DPIV-like activities was altered in the following manner. DP8/9 activity was decreased to 25 ± 5.0% ipsilateral versus 38 ± 4.5% contralateral, DPII activity was slightly increased to 59 ± 4.6% ipsilateral and 46 ± 5.0% contralateral, whereas fractional DPIV activity remained rather unchanged (11 ± 1.7%, ipsilateral versus 8 ± 2.2%, contralateral, Figure 4C). At the same time point, eMCAO induced a significant V_max _increase of DPII-specific activity (+237%) in ipsilateral cortices in comparison to the contralateral control tissue (p = 0.0001, Figure 4D, E). In parallel, we determined a rise of V_max _of DPIV activity (+262%) in ipsilateral cortices (*p *= 0.0004, Figure 4D, E).

**Figure 5:**
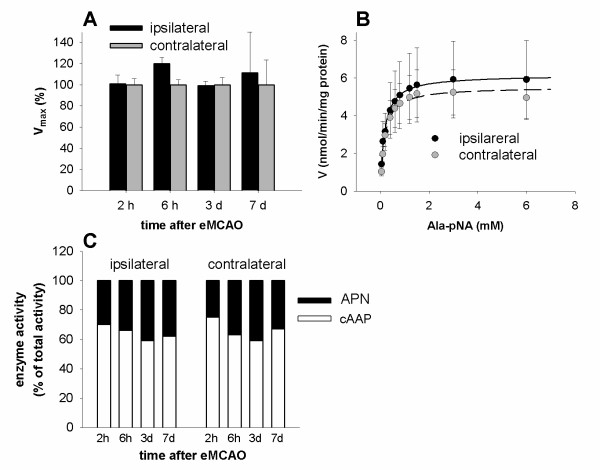
**APN and cAAP protease activities after transient cerebral ischemia**. (A) Relative V_max _of APN and cAAP activity in adult rat cortices of ipsilateral (infarct, black bars) or contralateral (control, gray bars) hemispheres at different time points after eMCAO. (B) Michaelis-Menten kinetics of total APN/cAAP activity in infarct (solid line) and control cortices (dashed line) at day 7 after eMCAO. Ipsilateral and contralateral cortices were characterized by roughly equal K_m _(infarct: K_m _= 160*μ*M ± 0.004*μ*M, n = 5; contralateral: K_m _= 160*μ*M ± 0.019*μ*M, n = 5) and similar V_max _values (infarct: 6.2 ± 2.1 nmol/min/mg protein, n = 5; contralateral: 5.5 ± 1.3 nmol/min/mg protein, n = 5). (C) Relative fractional contribution of APN (black) and cAAP (white) activity to total aminopeptidase activity (Ala-pNA hydrolysis) in the ipsilateral (infarct, left bar group) and contralateral (control, right bar group) hemisphere at different time points after eMCAO (pH **7**.5).

Similar to DPIV and DPIV-like proteases, the Michaelis-Menten-kinetics of APN and cAAP activity were studied in homogenates of ipsilateral (infarct) and contralateral (control) cortices after eMCAO. Instead of Gly-Pro-pNA, the hydrolysis of Ala-pNA was analyzed. From 2 h to day 7 post eMCAO, total activity of APN and cAAP remained unaffected (Figure 5A). Furthermore, ipsilateral and contralateral cortex homogenates were characterized by roughly equal K_m _(ipsilateral: K_m _= 160*μ*M ± 0.004*μ*M, n = 5; contralateral: K_m _= 160*μ*M ± 0.019*μ*M, n = 5) and similar V_max _values (ipsilateral: 6.2 ± 2.1 nmol/min/mg protein, n = 5; contralateral: 5.5 ± 1.3 nmol/min/mg protein, n = 5, Figure 5B). A competition study, using the selective cAAP inhibitor PAQ22 [20,28], revealed constant fractional activities of APN and cAAP in both hemispheres (Figure 5C).

### Effects of the non-selective DPIV and APN inhibitor IPC1755, and selective inhibition of DPIV, DPII or DP8/9 on ipsilateral lesion sizes after eMCAO

**Figure 6:**
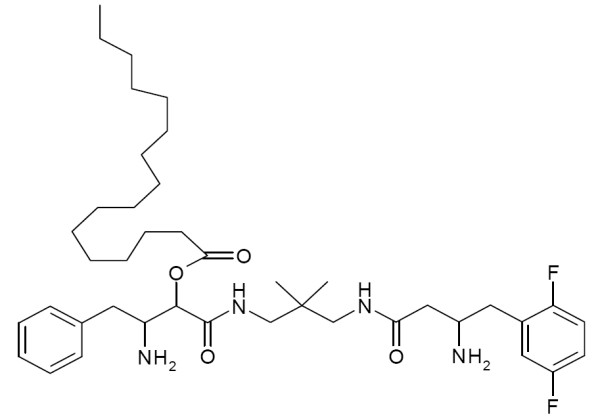
**Chemical structure of IPC1755**.

**Figure 7:**
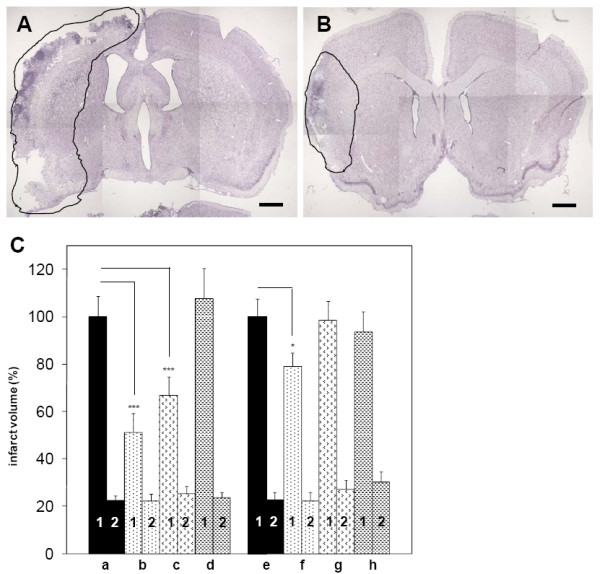
**Effect of non-selective or selective inhibition of DPIV and DPIV-like proteases on eMCAO-induced neurodegeneration**. Nissl-stained brain sections show cortical and striatal infarct areas 7 days post eMCAO, marked with black lines of one (A) vehicle- or (B) IPC1755-treated animal at anterior-posterior level Bregma: 0.40 mm to 0.80 mm. (A) and (B) illustrate infarct areas of individual brain sections corresponding to infarct volumes given in C, columns a and b, respectively. Scale bars = 1 mm (C) Morphometrical analysis of cortical and striatal infarct volumes. Equal volumes (2 μl) of vehicle (column a, e) or IPC1755, a non-selective inhibitor of DPIV/DPIV-like and APN/cAAP protease activity (columns b-d) or the specific DPIV inhibitor sitagliptin (column f) or selective inhibitors of DPII (column g) and DP8/9 (column h) were injected *icv *(f.c. 10 μM) according to the following time schemes. Columns a, b, e-h: first injection in parallel to eMCAO and subsequently, 6 and 24 h after eMCAO; column c: first injection 2 h after eMCAO and then, 6 and 24 h post eMCAO or column d: first injection 6 h and second injection 24 h after eMCAO. Infarct volumes were analyzed 7 days post eMCAO. Cortical (1) and striatal (2) lesion volumes of ipsilateral rat brain hemispheres were quantified individually. Data are given as relative values (% of control damage of vehicle-treated animals). In general, cortical infarct volumes of vehicle-treated control animals were set to 100% (column a, e). See Results for corresponding absolute infarct sizes (mm^3^). Data are given as means ± S.E.M., *n *= 8 - 12 animals per treatment group ***p ≤ 0.001 and *p ≤ 0.05, compared to vehicle-treated animals, *t*-test.

In order to address the question, whether dual inhibition of DPIV- and APN-like protease activities supports neuronal cell survival after transient cerebral ischemia, IPC1755, an inhibitor of DPIV/DPIV-like and APN/cAAP protease activity (Figure 6, see Materials for reference and protease-specific IC_50 _values), was administered *icv *ipsilateral to eMCAO. 7 days post insult, the infarct volume of vehicle (PBS)-treated control animals was 100.9 ± 8.9 mm^3 ^in the ipsilateral cortex, and 22.6 ± 2.4 mm^3 ^in the ipsilateral striatum (Figure 7A, C, column a). In the first series, IPC1755 (10 μM) was given in parallel to the induction of eMCAO and in addition, 6 h and 24 h post insult. Under these conditions, IPC1755 reduced the eMCAO-induced infarct volume in ipsilateral cortices by 48.8 ± 7.9% compared to vehicle-treated control animals (Figure 7B, C, column b). We still observed a significant reduction of eMCAO-induced lesion sizes by 33,0 ± 7.9%, when the first IPC1755 administration was delayed until 2 h post eMCAO (Figure 7C, column c). An IPC1755-dependent effect on lesion sizes was not observed, if IPC1755 was exclusively applied 6 and 24 h post eMCAO (107.5 ± 12.8%, Figure 7C, column d). These findings reveal a potential, therapy-relevant time window between 2 and 6 h post ischemia. In general, IPC1755 did not affect striatal lesion sizes (Figure 7C, columns b-d).

In the next series of experiments, we have investigated the influence of subtype-specific inhibition of DPIV, DPII or DP8/9 on neuronal cell survival after eMCAO. In analogy to IPC1755, each inhibitor (see Materials for details) was administered *icv *in parallel to the induction of eMCAO as well as 6 h and 24 h post insult (f.c. 10 μM for each injection). 7 days post insult, the infarct volumes of vehicle (PBS)-treated control animals were 132.2 ± 8.0 mm^3 ^and 29.9 ± 3.1 mm^3 ^in the ipsilateral cortex and striatum, respectively (Figure 7C, column e). According to the above application protocol, only sitagliptin, a selective DPIV inhibitor, was able to reduce the cortical infarct size by 21.1 ± 5.8% compared to vehicle (Figure 7C, column e, f). This neuroprotective effect of sitagliptin was smaller than the effect of the non-selective protease inhibitor IPC1755 using the same experimental conditions (48.8 ± 7.9%, compare Figure 7C, column a, b, e, f). In contrast to sitagliptin, selective DPII or DP8/9 inhibition did not decrease eMCAO-induced infarct volumes in ipsilateral cortices (Figure 7C, column e, g, h). Similar to IPC1755, neither sitagliptin nor selective DPII or DP8/9 inhibition affected striatal lesion sizes (Figure 7C, columns a, b, e-h).

## Discussion

DPIV, APN as well as DPIV- and APN-like proteases are known to regulate T cell activation and/or the synthesis and the release of certain cytokines, chemokines and neurotransmitters. Moreover, inhibitors of DPIV and APN have been frequently considered as potential candidates for the therapy of autoimmune diseases and post-transplantation immune reactivity. Behind this, ischemia-induced neuronal cell degeneration and cerebral damage, for instance due to a stroke, traumatic brain injury or cardiac arrest, involves massive immune reactions in the brain which promote a further, secondary brain damage [2]. Based on this deleterious cascade, the reduction and control of cerebral inflammation after cerebral ischemia can be considered as valid therapeutic option to improve neuronal cell survival and the clinical outcome of these patients.

Although mRNA expression of DPIV and DPIV-like proteases was previously reported in the brain [21-25], the regulation of these targets in acute, cerebral ischemia-triggered neuronal degeneration and inflammation has remained widely unknown.

As reported here, mRNA expression of DPIV, DPII, DP8, DP9, APN and cAAP is localized in ipsilateral and contralateral brain hemispheres. After transient and unilateral cerebral ischemia, protease-specific mRNA expression was differentially affected in the ipsilateral cortex. DPII and APN were continuously up-regulated from 6 h to 7 days post eMCAO. In contrast, DP9 and cAAP were transiently down-regulated, whereas DPIV remained unchanged.

Protease-specific mRNA expression and protein localization patterns did not overlap entirely. In particular, DPIV-specific immunoreactivity was generally not detectable in brains of control animals and in the contralateral hemisphere after unilateral induction of eMCAO. Contrarily, DP8 and APN immunoreactivities were localized in contralateral cortices, i.e. in nuclei of intact cortical neurons.

Notably, the appearance of DPIV-linked immunoreactivity in the rat brain was ischemia-dependent and exclusively restricted to the ipsilateral hemisphere, arising first in immune reactive microglia (3 d post eMCAO) and subsequently spreading to activated astroglial cells and surviving neurons (7 d post eMCAO). Despite constantly detectable DPIV mRNA levels in post-ischemic and non-ischemic cortices, immunoreactivity of this protease was ischemia-dependent. In the ipsilateral cortex, also DP8 and APN immunoreactivties were affected by eMCAO, i.e. with a similar temporal, but distinct spatial pattern. Both proteases were targeted in immune-reactive macrophages and astroglia 3 and 7 days post eMCAO, respectively. These findings suggest the involvement of DPIV, DP8 and APN in the regulation of immune-competent cells and intracerebral inflammation in response to cerebral ischemia. The pattern of mRNA expression and immunohistochemical detection of DP8 was similar to DPIV, i.e. we found a cell-type specific and time-dependent detection of DP8 in reactive microglia/macrophages (3 days post eMCAO) and astroglia (7 days post eMCAO) without changes in mRNA expression.

In addition, surviving neurons in the ipsilateral, ischemia-exposed cortex were characterized by DPIV-positive perikarya and DP8- and APN-positive nuclei 7 days post eMCAO. These results indicate a role of these proteases in ischemia-dependent neuronal signalling cascades. Nevertheless, it remains to be clarified, whether their appearance is linked to deleterious or beneficial effects in neurons surviving the eMCAO-induced ischemia.

In adult rat brains, functionally active DPII, DP8/9, APN and cAAP were identified in cerebral cortices of both brain hemispheres, independent of the exposure to eMCAO. These findings were consistent with our RT-PCR and immunohistochemistry results. Low levels of DPIV activity were found also in contralateral cortices of adult rats. The latter finding was in line with the expression pattern of DPIV, but was not supported by immunohistochemistry.

Seven days after the induction of eMCAO, a significant increase of DPIV and DPIV-like activity, i.e. V_max_, was discovered. As revealed by fractional protease activity studies, DPII, DPIV and, to a smaller extent, DP8/9 contributed to the ischemia-triggered rise of protease activity in ipsilateral cortices 7 days post eMCAO. Over the first 7 days after eMCAO, the ratio of DPII/DP8/9 activity increased continuously in the ipsilateral cortex, but remained constant contralateral. In general, protease activities of APN and cAAP were not altered over the first 7 days post eMCAO. Rather, V_max _and K_m _values of both enzymes as well as the ratio of fractional APN and cAAP activities remained constant.

Ischemia-dependent activity patterns of DPIV- and DPIV-like proteases were at least partly mirrored by their temporal expression pattern. For instance, the continuous rise of DPII activity over the first 7 days post eMCAO was coincident with the increase of DPII-specific mRNA obtained in the qualitative RT-PCR analysis. Furthermore, the decreasing contribution of DP8/9 to total DPIV-like activity was roughly in line with constant or transiently decreased mRNA levels of DP8 and DP9, respectively, in the ipsilateral cortex. On the other hand, it remains unclear, how eMCAO-independent activities of APN and cAAP correspond to the up-regulation of APN and transiently decreased cAAP mRNA levels in the ipsilateral hemisphere.

Our findings suggest that DPII, DPIV, DP8 and DP9 as well as APN and cAAP may be directly linked to the activation and proliferation of brain-innate immune-competent cells after eMCAO and a transient cerebral ischemia. Conceivably, increased protease activities emerge at early time points after ischemic insults. This study suggests that DPIV- and APN-like protease immunoreactivities, except DPIV itself, are mainly assigned to neuronal cells in unaffected, healthy brain areas. In addition, increased activities of DPIV and DPIV-like enzymes coincide with the vast proliferation and activation of microglia and macrophages around day 3 after eMCAO. Thus, increased protease activity induced by glia activation may counteract the decrease of neuronal proteases resulting from neuronal cell death during the first days post insult. Furthermore, astroglia activation in the ipsilateral cortex at later time points could underlie the significant increase in the sum protease activity after eMCAO. Due to the decline of vital neurons until day 3 post insult, a strongly decreased protease immunoreactivity of neurons was detected. In parallel, there was a robust co-localisation of DPIV/APN proteases with microglial markers. In the contralateral hemisphere, microglia activation as well as co-staining of DPIV, DP8 and APN and microglia markers was not observed.

As known, neuronal degeneration lasts over the first hours post ischemia, whereas microglia activation is delayed and reaches its maximum around day 3 post insult. Subsequently, the number of activated microglia cells pulls back to nearly baseline values, but with concomitant astroglia proliferation and activation. This delayed activation pattern appears to be typical for ischemia-induced brain damage [2]. The present study reveals now that the temporal regulation of glia cell type proliferation and activation is accompanied by the specific regulation and activation of DPIV, APN and distinct DPIV- and APN-like proteases. Notably, neuron-specific DPIV was exclusively identified in surviving neurons at day 7 post eMCAO. In addition, DPIV was a characteristic feature of activated microglia, macrophages and astroglia in the ipsilateral cortex 3 and 7 days post eMCAO, respectively. A similar or even more pronounced pattern was obtained with respect to DP8/9 and DPII. DPII as well as DP8/9-specific protease activity were potently caused by glia proliferation and activation within the first days post eMCAO.

The involvement of DPIV, APN and DPIV-/APN-like proteases in ischemia-triggered signaling cascades was confirmed by the ability of the non-selective inhibitor of DPIV and APN protease activity IPC1755 (10 μM) to reduce cortical lesion sizes and to promote neuronal cell survival after a transient focal ischemia due to eMCAO.

**Figure 8:**
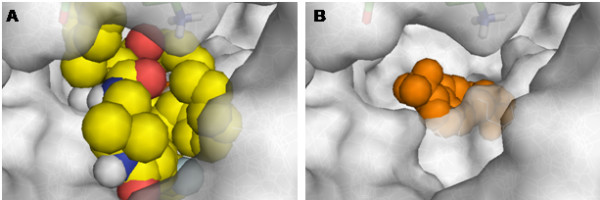
**Inhibition of membrane-bound DPIV by IPC1755 and sitagliptin**. (A) IPC1755 (yellow, orange and blue marking) binds to the central pore binding site 2 nm in front of the active site of DPIV. Consequently, the central pore becomes completely blocked. This is crucial, because, in contrast to soluble DPIV, this pore remains the only substrate and inhibitor access route of the membrane-bound enzyme. In contrast to soluble DPIV, alternative substrate access through the side pore of the protease (not shown) has been postulated to be restricted by other membrane proteins, glycolipid-enriched domains and/or lipid rafts [17]. (A) Sitagliptin (orange) binds directly to the active site of DPIV and is therefore able to inhibit both, membrane-bound and soluble DPIV.

Based on the corresponding IC_50 _values of IPC1755, DPIV (0.06 μM), APN (0.03 μM) and/or cAAP (0.02 μM), rather than DPII (166 μM) or DP8/9 (85 μM), might serve as its potential targets in cerebral ischemia. This assumption was further supported by the finding that in contrast to selective blockers of DPII or DP8/9, sitagliptin (10 μM), a selective DPIV inhibitor, was also able to mediate neuroprotection, although to a smaller extent than IPC1755. Sitagliptin was the first DPIV inhibitor that was approved for the treatment of diabetes mellitus type-2. DPIV inhibitors mediate an anti-hyperglycemic effect by counteracting the proteolytic cleavage of incretins such as glucagon-like peptide-1 (GLP-1) and consequently, stimulate insulin and suppress glucagon release [29]. DPIV is characterized by a central pore that is crucially involved in the autosterical regulation and substrate access to the active site of the plasma membrane-bound protease [17]. Evidence for a similar regulation of APN is given. Synthetic inhibitors of membrane-bound DPIV able to suppress DNA synthesis and cytokine production of immune cells are either (1) ligands of a central pore binding site 2 nm apart from the active site, thereby blocking substrate access to the active site or (2) inhibitory ligands of the active site itself [17]. IPC1755 fits into the first paradigm (Figure 8A), whereas sitagliptin belongs to the second group of DPIV inhibitors (Figure 8B). In here, we provide experimental evidence suggesting that both modes of inhibition of membrane-bound DPIV can mediate neuronal protection after cerebral ischemia. Blockage of substrate access to the active site through the central pore of DPIV (by IPC1755) or alternatively, inhibition of the active site itself (by sitagliptin) were able to support neuronal survival after cerebral ischemia. Neuroprotection may result from the inhibition of brain-derived and peripheral immune cells circulating in the brain and consequently, the repression of devastating cerebral inflammation and secondary brain damage. In addition, DPIV inhibition may prevent the degradation of neuroprotective and/or anti-inflammatory peptides (e.g. neuropeptide Y, brain-derived neurotrophic factor and transforming growth factor ß). In this context, DPIV, APN and/or cAAP may act in a synergistic manner.

(1) Temporal protease activity profiles post eMCAO and (2) given protease specificities of IPC1755 and sitagliptin implicate that early inhibition of membrane-bound DPIV (by IPC1755 or sitagliptin), APN and/or cAAP (by IPC1755) induces anti-inflammatory and neuroprotective effects in the penumbra surrounding the infarct core and supports long-term neuronal survival after cerebral ischemia.

Notably, IPC1755 mediated neuronal protection, even if applied at delayed time points after eMCAO. A time window of 2 to 6 h between cerebral ischemia and treatment start might be appropriate for potential therapeutic approaches. It is well established that severe cerebral ischemia starts to trigger massive inflammation due to microglial activation and leukocyte infiltration within a few hours post insult. Four to six hours after ischemia, astrocytes become hypertrophic while miccroglial cells retract their processes and assume an amoeboid morphology that is typical of activated microglia [30]. We suggest that inhibition of DPIV, APN and/cAAP within a narrow time window may reduce crucial inflammation and protect vulnerable neurons within the ischemic penumbra. Additional treatments with IPC1755, for instance given 6 hour and 24 hour post ischemia, may provide further support although being insufficient to promote neuroprotection if given exclusively. Nevertheless, further studies are required to identify most suitable drug application protocols for preventive and/or therapeutic intervention strategies.

## Conclusions

Taken together, this study suggests distinct inflammatory regulator proteases as potentially important mediators of ischemia-induced neuronal cell death and cerebral inflammation. This may imply new options to fight the deleterious consequences of cerebral ischemia and/or deregulated inflammation in the context of acute and chronic neurodegeneration.

## Methods

### Animals

Studies were performed on male Sprague-Dawley rats (250-280 g) obtained from Harlan Winkelmann (Borchen, Germany). Animals were maintained under constant environmental conditions with an ambient temperature of 21 ± 2°C and relative humidity of 40%. They were housed with a 12-h light-dark cycle, food and water were given ad libitum.

All animal experiments described in this study were in accordance with the legal requirements of the German Tierschutzgesetz from 1998. The study was approved by the authorities of the State of Saxony-Anhalt (Landesverwaltungsamt) and performed according to institutional guidelines.

### Materials

L-2,4-Diaminobutyrylpiperidinamide, a selective inhibitor of DPII [31], was purchased from Merck/Calbiochem (Darmstadt, Germany). (2*S*,3*R*)-2-(2-amino-3-methyl-1-oxopentan-1-yl)-1,3-dihydro-2*H*-isoindole hydrochloride as selective DP8/DP9 inhibitor and (2*S*)-2-[4-[[[[(2*S*)-1-[(3*R*)-3-amino-4-(2,5-difluorophenyl)-1-oxobutyl]-2-pyrrolidinyl]carbonyl]amino]methyl]phenoxy]-3-methylbutanoic acid, trifluoroacetate as a selective DPIV-inhibitor were synthesized according to [32]. Sitagliptin ((R)-3-Amino-1-[3-(trifluormethyl)- 5,6,7,8-tetrahydro[1,2,4]triazol[4,3-a]pyrazin-7-yl]- 4-(2,4,5-trifluorphenyl)butan-1-on), a selective DPIV inhibitor was purchased from Merck Sharp & Dohme (Pavia, Italy). Actinonin, an inhibitor of APN and cAAP, was purchased from Sigma (Taufkirchen, Germany), and PAQ-22, a specific inhibitor of cAAP, was synthesized according to [28].

IPC1755 was synthesized by MOLISA (Magdeburg, Germany). IPC1755 (25 mmol) was dissolved in dimethyl sulfoxide (DMSO, Sigma-Aldrich, Germany) and diluted with 0.1 M phosphate buffer saline (PBS, pH 7.4) to a final stock concentration of 10 mmol/l. Sitagliptin, L-2,4-Diaminobutyrylpiperidinamide (a selective DPII inhibitor) or (2*S*,3*R*)-2-(2-amino-3-methyl-1-oxopentan-1-yl)-1,3-dihydro-2*H*-isoindole hydrochloride (selective DP8/9-inhibitor) were separately diluted with 0.1 M phosphate buffer saline (PBS, pH 7.4) to a final stock concentration of 10 mmol/l. Inhibitor-specific stock solutions (2 μL) were administered intracerebroventricularly (*icv*) at different time points after the induction of eMCAO (final drug concentrations 10 μM). IPC1755 (Patent EP09169269) is a non-selective DPIV and APN inhibitor with the following values of protease-specific half maximal inhibitory concentrations (IC_50_): 0.06 μM (DPIV), 0.03 μM (APN), 0.02 μM (cAAP), 166 μM (DPII) and 85 μM (DP8/9). IPC1755 suppressed the proliferation of mononuclear cells (IC_50 _= 36 μM) and T cells (IC_50 _= 34 μM).

All other chemicals were of highest available purity.

### Induction of eMCAO

Procedures were designed such that experimental animals do not suffer unnecessarily. eMCAO was performed according to Sharkey and Butcher [33] with the following modifications. Animals were anesthetized with halothane in a mixture of nitrous oxide/oxygen (70:30) and maintained with 2-3% halothane during the following procedures: rats were placed in a Kopf stereotaxic frame and further anesthetized via a nose cone. For the induction of eMCAO, a burr hole (1 mm diameter) was drilled into the skull (coordinates: anterior 0.50 mm from bregma, lateral 5.2 mm to satura sagittalis). After careful opening of the dura, a 29-gauge cannula was lowered 7.5 mm below the dura according to the rat brain atlas of Paxinos and Watson [34]. To induce eMCAO, rats received an injection of 60 pmol of endothelin 1 (ED-1, Sigma-Aldrich) in 3 μl of 0.1 M phosphate-buffered saline, pH 7.4, over a time period of 5 min. After further 5 min, the cannula was slowly withdrawn. Throughout the operation procedure, rats were kept at 37 ± 0.5°C using a thermostatically controlled heating blanket attached to a rectal thermometer. The animals were then placed under a heating lamp to maintain normothermia until recovery.

### Preparation of total RNA

Cortical tissue probes from ipsilateral and contralateral brain hemispheres were prepared separately at defined time points after eMCAO and then pounded with a pestle and mortar under liquid nitrogen to a fine powder. After homogenization in TRIzol reagent (Invitrogen, Darmstadt, Germany), 0.2 ml chloroform were added, the samples were extensively shaken by hand and then incubated at room temperature for 2 min. Subsequently, the tissue suspensions were centrifuged for phase separation at 12,000 × g at 4°C for 10 min. The upper RNA-containing aqueous phase was collected, mixed with the 0.7-fold volume of isopropanol and incubated at room temperature for 10 min. The precipitated RNA was collected by centrifugation with 12,000 × g at 4°C for 10 min. The RNA was washed with 75% ethanol and again centrifuged at 7,500 × g at 4°C for 5 min. The supernatant was carefully removed and the pellet air-dried at room temperature for 10 min. The total RNA was then resolved in 100 μl RNase-free water. The RNA concentration was determined by UV spectroscopy, using a GeneQuant spectrophotometer (Amersham Biosciences, Freiburg, Germany). RNA integrity was checked in agarose gels.

### Reverse transcription of total RNA

After 5 min pre-incubation of 2.5 μg total RNA with random hexanucleotides (Boehringer, Mannheim, Germany) in a final concentration of 4 mM at 70°C, the supplied avian myeloblastosis virus (AMV) reverse transcriptase buffer, desoxynucleoside triphosphates in a final concentration of 1 mM and 50 units of RNAsin ribonuclease inhibitor (Promega, Mannheim, Deutschland) were added. Samples were cooled down to 25°C before 20 units of AMV reverse transcriptase (Promega, Mannheim, Germany) were added yielding a final volume of the reaction mixture of 20 μl. After 10 min at 25°C, reverse transcription was performed at 42°C for 1 h. The enzymatic reaction was stopped by a 10 min incubation at 70°C.

### PCR

PCR was carried out with 125 ng cDNA using a PCR-Hot Start-Mix Y (PeqLab, Erlangen, Germany). The reaction mixture (final volume 50 μl) contained 10 μM of protease and rat-specific primers. The temperature program was performed in a lid heated thermal cycler (Bio-Rad, Munich, Germany), started by the initial activation at 95°C for 15 min. A primer-dependent number of PCR cycles was consisting of denaturation at 94°C for 30 s, annealing at 55°C for 30 s and elongation at 72°C for 30 s. PCR-products were analyzed with a CCD camera-equipped UV transilluminator after electrophoresis in agarose gel.

### Immunohistochemistry

At different time points after eMCAO, rats were deeply anesthezised and the brains were fixed with 4% paraformaldehyde (PFA) in 0.1 M phosphate buffer by a transcardial perfusion. Subsequently, the brains were removed, dissected, postfixed in 4% PFA/PBS overnight at 4°C and placed in a rat brain matrix (ASI Instruments, Warren, MI, USA). For the immunohistochemical localisation of DPIV, DP8 and APN in specific brain cell types, 1 mm thick coronal brain slices were cut with a razor blade at 14 pre-determined anterior-posterior levels comprising the cortical infarct area. After cryoprotection with 30% sucrose, adjacent cryostat sections (20 μm) of each brain slice were cut in a Cryo-Star HM560M microtome (Microm International, Walldorf, Germany) and washed in PBS. Free floating sections were permeabilized in 0.1 M PBS containing 0.5% Triton X-100 for 30 min followed by incubation in blocking serum (10% goat normal serum in 0.1 M PBS for 30 min). Subsequently, the sections were immunostained with the respective primary antibodies, i.e. monoclonal mouse anti-rat DPIV (OX61, CD26, 1:500; Abcam, Cambridge, UK), polyclonal rabbit anti-DP8 (1:500; Abcam) or polyclonal rabbit anti-APN (CD13, 1:50; Santa Cruz Biotechnology, Santa Cruz, CA, USA). For double labeling, these antibodies were incubated simultaneously with specific cellular markers, i.e. a monoclonal mouse anti-neuronal nuclei (NeuN, 1:500, Millipore-Chemicon, Billerica, MA, USA) to identify neurons, a monoclonal mouse anti-glial fibrilary acid protein (GFAP, 1:200, Molecular Probes, Leiden, The Netherlands) to target astroglia and a monoclonal mouse anti-rat monocytes/macrophages (ED1, CD68, 1:500, Millipore-Chemicon) or IB4-Alexa Fluor 488 (isolectin from Griffonia simlicifolia, Invitrogen, Darmstadt, Germany)) to label microglia. Tissue sections and antibodies were incubated at 4°C over night. For immunofluorescence visualization, sections were incubated with fluorescence-labeled secondary antibodies at room temperature for 2 h, i.e. Alexa Fluor 488 goat anti-mouse IgG (1:200; Molecular Probes) and Alexa Fluor 594 goat anti-rabbit IgG (1:200, Molecular Probes). In order to reveal the cytoarchitecture of brain sections, a Nissl staining with Neurotrace fluorescence (Molecular Probes) was performed. Sections were washed several times, mounted onto glass slides and embedded with Vectashield containing DAPI to stain nuclei. The specificity of all antibodies used was assessed by identical immunofluorescence staining procedures, with the only exception that the respective primary antibodies were omitted. Colocalisation studies of DPIV, DP8/9 or APN with specific cell markers were analyzed using a confocal laser scanning fluorescence microscope (LSM 5 Pascal, Carl Zeiss, Jena, Germany).

### Measurement of DPIV protease activity (H-Gly-Pro-pNA hydrolysis)

Protease activity measurements were performed in tissue homogenates freshly prepared from rat cortex. Cortices from ipsilateral or contralateral hemispheres after eMCAO or from control animals were separately prepared and analyzed. Animals were sacrificed by cervical dislocation. Cortices were dissected, wet weight measured and subsequently, roughly chopped with scissors and transferred into a tissue potter containing 1 ml PBS with 1% Triton X-100. The homogenization was performed by rotary and vertical potter movements. The homogenates were diluted in PBS to a final concentration of 10 mg wet weight/ml.

A continuous fluorimetric assay was performed by applying distinct concentrations of H-Gly-Pro-pNA as substrate of DPIV and DPIV-like proteases (DPII, DP8 and DP9). The liberation of p-nitro-aniline (pNA) was measured in a total volume of 200 μl PBS (10 mM substrate, pH 7.5, Sigma-Aldrich, Taufkirchen, Germany) containing 80 μl tissue homogenate. The pNA absorption was measured at 390 nm using a Microplate-Reader (FluoStar Optima, BMG-Labtech, Offenburg, Germany). Protease activity, expressed as nmol/min/mg protein, was calculated using a pNA-standard curve. Protein concentrations were quantified using the BCA Protein Assay Kit (Pierce Biotechnology, Rockford, IL, USA).

In general, ipsilateral and contralateral cortices were analyzed separately at pre-defined time points after eMCAO. Fractional activities of DPIV and the different DPIV-like proteases were calculated from protease-specific inhibitor titration curves that were individually generated with a selective inhibitor of DPII, DPIV or DP8/9 (final inhibitor concentration 10^-12 ^M to 10^-3 ^M) and Gly-Pro-pNA (400 μM) at pH 7.5 (for details, see Calculations).

### Measurement of APN enzymatic activity (Ala-pNA hydolysis)

APN and cAAP protease activity was determined using Ala-pNA (800 μM) as substrate and the same assay conditions as described for DPIV measurements. Competitive inhibition curves were obtained by the adjustment of increasing concentrations of the specific cAAP inhibitor PAQ22 and the non-selective APN/cAAP inhibitor actinonin, respectively (final concentration of each inhibitor 10^-12 ^M to 10^-4 ^M). The fractional activity of cAAP in homogenates was calculated from the maximum PAQ22-inhibited enzyme activity as percent of total actinonin-sensitive protease activity at pH 7.5.

### Calculations

For data analysis, SigmaPlot and SigmaStat software packages were used (Systat Software, Erkrath, Germany). The kinetic data analysis was performed using the Michaelis-Menten-equation. For the analysis of fractional protease activities, subtype-selective inhibitor titration curves were fitted using the equation for a two-side competition curve:

$$y=\min+\left[(\max-\min)*\left(\frac{F1}{1+10^{\left(x-\log IC_{50}1\right)}}\right)\right]+\left(\frac{\left(1-F1\right)}{1+10^{\left(x-\log IC_{50}2\right)}}\right)$$

IC_50_1   IC_50_-value of the inhibitor (high affinity inhibition)

IC_50_2   IC_50_-value of the inhibitor (low affinity inhibition)

F1   ratio of inhibition side (high affinity inhibition)

max   maximum activity (without inhibition)

min   minimum activity (maximum inhibition)

x   given inhibitor concentration as decadic log (variable)

y   product concentration (dependent variable)

### Effect of protease inhibitors on infarct volumes after eMCAO

In order to perform the *icv *application of different protease inhibitors under halothane anesthesia, a second burr hole was drilled into the skull (coordinates: posterior 0.80 mm from bregma, lateral 1.5 mm to satura sagittalis). Then, a 29-gauge cannula was lowered 4.5 mm below the dura. The *icv *injection of IPC1755 (2 μl of a 10 mmol/l PBS/DMSO solution, pH 7.4, f.c. 10 μM) was performed according to one of the following time schedules; (1) during ischemia, 6 and 24 h after eMCAO, (2) 2, 6, and 24 h after eMCAO and (3) only 6 and 24 h after eMCAO. Sitagliptin as well as DPII- or DP8/9-specific inhibitors (2 μl of a 10 mmol/l PBS/DMSO solution, pH 7.4, f.c. 10 μM) were administered exclusively according to schedule 1, i.e. during eMCAO, 6 and 24 h after eMCAO. For controls, vehicle (2 μl) instead of the corresponding protease inhibitor was injected. According to legal requirements of the German animal law, animals were kept under anesthesia during each drug injection. Control rats were treated in the same way, except that the corresponding vehicle solution instead of the drug was injected. Thus, potential protective effects of halothane can be excluded. Rats were maintained at 37 ± 0.5°C throughout the operation procedures. In parallel, the body temperature was monitored using a rectal temperature sensor. Subsequently, the animals were placed into an incubator to maintain normothermia until recovery from anesthesia and then returned into their home cage.

After a survival time of 7 days after eMCAO, animals were anesthetized by intraperitonial injection of pentobarbital and transcardially perfusion-fixed with saline followed by 4% paraformaldehyde in 0.1 M PBS (pH 7.4). Brains were then removed carefully, post-fixed in the same fixative for 2 h, and placed in a rodent brain matrix (rat, ASI Instruments, Warren, MI, USA). 1-mm coronal brain slices were cut with a razor blade at 14 predetermined anterior-posterior levels. After cryoprotection in 30% sucrose, slices were rapidly frozen in isopentane and stored at -80°C. Four to five cryostat sections (30 μm) from each brain slice were cut in a cryo microtome and stained with toluidine blue.

The extent of cortical and striatal damage following ischemic injury was documented with microphotographic images from Nissl-stained slices showing the anterior-posterior level according to the brain atlas of Paxinos and Watson [34]. The volumes of cortical and striatal infarct lesions were measured at eight predetermined anterior-posterior levels by an operator blinded to the group composition. Three-dimensional lesion sizes were calculated by integration of two-dimensional lesion sizes at each stereotactic level and the distances between the various levels. Image analysis was performed with an Eclipse TE 3000 microscope (Nikon, Düsseldorf, Germany), equipped with a 4x objective, and the Lucia software package (Version 4.2.1., Nikon). Data were statistically analyzed by non-paired Student's *t*-test and given as mean ± S.E.M. Statistical significance was defined at *p *values < 0.001 or *p *< 0.01 as indicated.

## Abbreviations

APN: Aminopeptidase N; cAAP: Cytosolic alanyl-aminopeptidase; DPIV: Dipeptidyl peptidase IV; DPII: Dipeptidyl peptidase II; DP8: Dipeptidyl peptidase 8; DP9: Dipeptidyl peptidase 9; eMCAO: Endothelin-induced occlusion of the middle cerebral artery; GLP-1: glucagon-like peptide-1; GIP: Glucose-dependent insulinotropic polypeptide; icv: Intracerebroventricular; pNA: Para-nitro-aniline.

## Competing interests

PR, WS, PE and FS were employees of KeyNeurotek Pharmaceuticals until June 30, 2010. Due to a changed strategic focus, the company cancelled all research and development activities related to DPIV and APN in March 2010. UB, KN, MT and SA are employees of IMTM GmbH. IMTM holds and has applied for patents relating to the design and/or clinical use of protease inhibitors including DPIV and APN. PR, FS, UB, KN, MT and/or SA are co-inventors of those patents and patent applications.

## Authors' contributions

PR contributed to the conception of the study, data analysis and interpretation. He designed, carried out and analyzed the protease activity assays, was involved in the design of IPC1755 and drafting the manuscript. WS contributed to the conception of the study, data analysis and interpretation. He designed, performed and analyzed the in vivo experiments including immunohistochemistry and was involved in drafting the manuscript. PE performed and analyzed the protease activity assays. AG and SW performed and analyzed RT-PCR experiments and provided critical input to the manuscript. UB participated in the molecular design of IPC1755, the development and design of protease assays and data discussion. KN was involved in the design of IPC1755 and data interpretation. MT and SA led the development of IPC1755 and participated in data analysis and drafting the manuscript. DR contributed to the conception, design and interpretation of the study, supervised the RT-PCR experiments and was involved in drafting the manuscript. FS contributed to the conception, design and interpretation of the study, participated in the design of IPC1755 and wrote the manuscript. All authors read and approved the final manuscript.
